# Advancing Cerebro-cerebellar Network Imaging with Ultra-high Field (7T) MRI: Progress and Future Challenges

**DOI:** 10.1007/s12311-026-02054-7

**Published:** 2026-07-25

**Authors:** Amina Zohor Zidane Burgess, Jackson Tyler Boonstra, Yoshifumi Mori, Sheeba Anteraper, Wietske van der Zwaag

**Affiliations:** 1 Netherlands Institute for Neuroscience, Royal Netherlands Academy of Arts and Sciences, Amsterdam, the Netherlands; 2 Faculty of Behavioural and Movement Sciences, Vrije Universiteit Amsterdam, Amsterdam, the Netherlands; 3 Department of Neurology, Amsterdam University Medical Center, Amsterdam, the Netherlands; 4 Spinoza Centre for Neuroimaging, Amsterdam, the Netherlands; 5 Advanced Imaging Research Center, University of Texas Southwestern Medical center (UTSW), Dallas, TX USA

**Keywords:** Ultra-high field MRI, Cerebro-cerebellar networks, Cerebellum

## Abstract

Ultra-high field (UHF) MRI (≥ 7T) is increasingly used to study the cerebro-cerebellar networks, offering substantial gains in signal-to-noise ratio and blood oxygen level dependent (BOLD) sensitivity that enable submillimeter functional and structural imaging. These advantages are particularly relevant for the cerebellum, whose tightly folded cortex and small deep nuclei have historically been difficult to resolve *in vivo*. In this targeted review, we synthesize recent progress in UHF-MRI applied to cerebro-cerebellar network mapping and critically examine the methodological challenges that accompany these developments. We highlight how UHF-MRI has enabled more precise delineation of cerebellar functional territories, improved visualization of the dentate nucleus and its connectivity, and facilitated integration of cerebellar nodes into whole-brain network models. We also discuss the technical challenges, including RF-field inhomogeneity, susceptibility-induced distortions, and the trade-off between spatial resolution and signal strength. These issues are compounded in the cerebellum due to its anatomical location and fine-scale organization, increasing the risk of spatial biases and misinterpretation if not carefully addressed. Beyond acquisition, we identify key gaps in analysis methodologies, including the need for cerebellum-specific preprocessing pipelines, segmentation tools that preserve mesoscale anatomy, and frameworks that treat the cerebellum and cerebrum as a unified system. We also discuss emerging opportunities, such as neurostimulation, integration with postmortem imaging and deep learning-based methods that may help bridge microstructural and *in vivo* findings. Overall, we argue that while UHF-MRI holds transformative potential for cerebellar systems neuroscience, translational progress will depend on rigorous methodological standardization and awareness of its limitations.

## The Cerebro-cerebellar Network

**Fig. 1:**
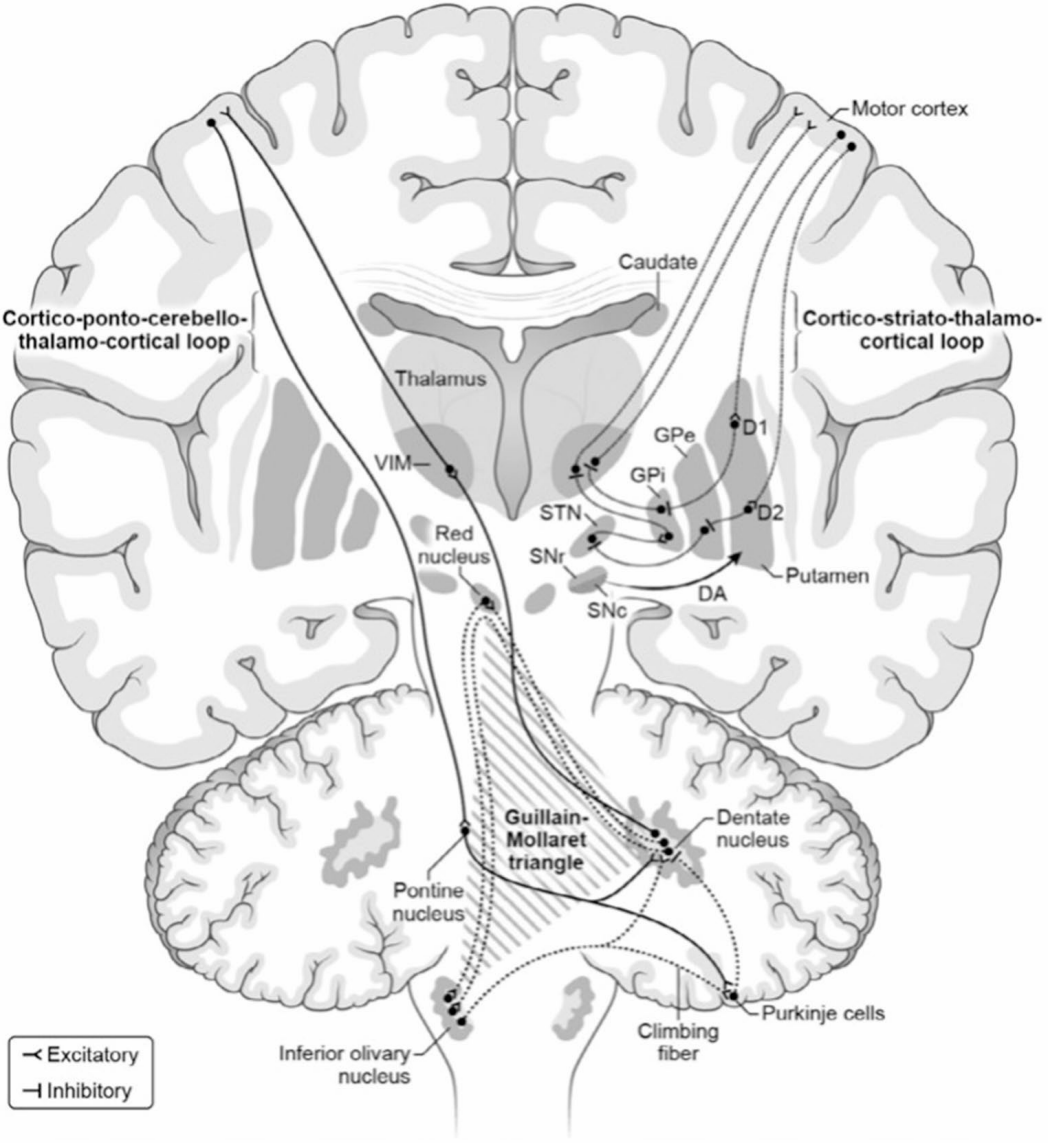
Schematic illustration of the brain regions and pathways of the cerebro-cerebellar network. Adapted from [1]

The cerebellar and cerebral cortices are part of an extensive cerebro-cerebellar network. The cerebellum receives input from the body through the inferior olive in the brainstem, from where climbing fibres pass through the inferior cerebellar peduncles to project to the Purkinje cells in the cerebellar cortex (Fig. 1) [1, 2]. Output from the cerebellar cortex passes through the cerebellar dentate nucleus (DN) and thalamus to the cerebral cortex along the dentatothalamic tract. Feedback from the cerebral cortex makes its way back to the cerebellum through the pons along the mossy fibres which enter the cerebellum via the middle and inferior cerebellar peduncles, closing the cerebro-cerebellar loop [2, 3]. All known networks in the brain contain these loops, and each part of the cerebellar cortex is linked to a specific cerebral cortical area through cerebro-cerebellar loops [4]. In humans, both resting-state and task-based fMRI at conventional field strengths have demonstrated that the DN and cerebellar cortex are functionally connected with widespread cerebral regions, including motor, prefrontal, and parietal association areas [5–7]. Motor networks map on to roughly one-third of the cerebellar cortical area, whereas the fronto-parietal networks map on to larger cerebellar areas, highlighting the cerebellum’s role in cognition and affect [8]. Using ultra-high field (UHF, ≥7T) MRI, the nodes of these extensive networks can be visualised and probed to reveal their inner functional organisation. In this targeted review, we will highlight the progress and challenges associated with UHF-MRI, and provide directions for future research.

## Technical Advantages and Challenges for UHF-MRI in Cerebro-cerebellar Network Imaging

**Fig. 2:**
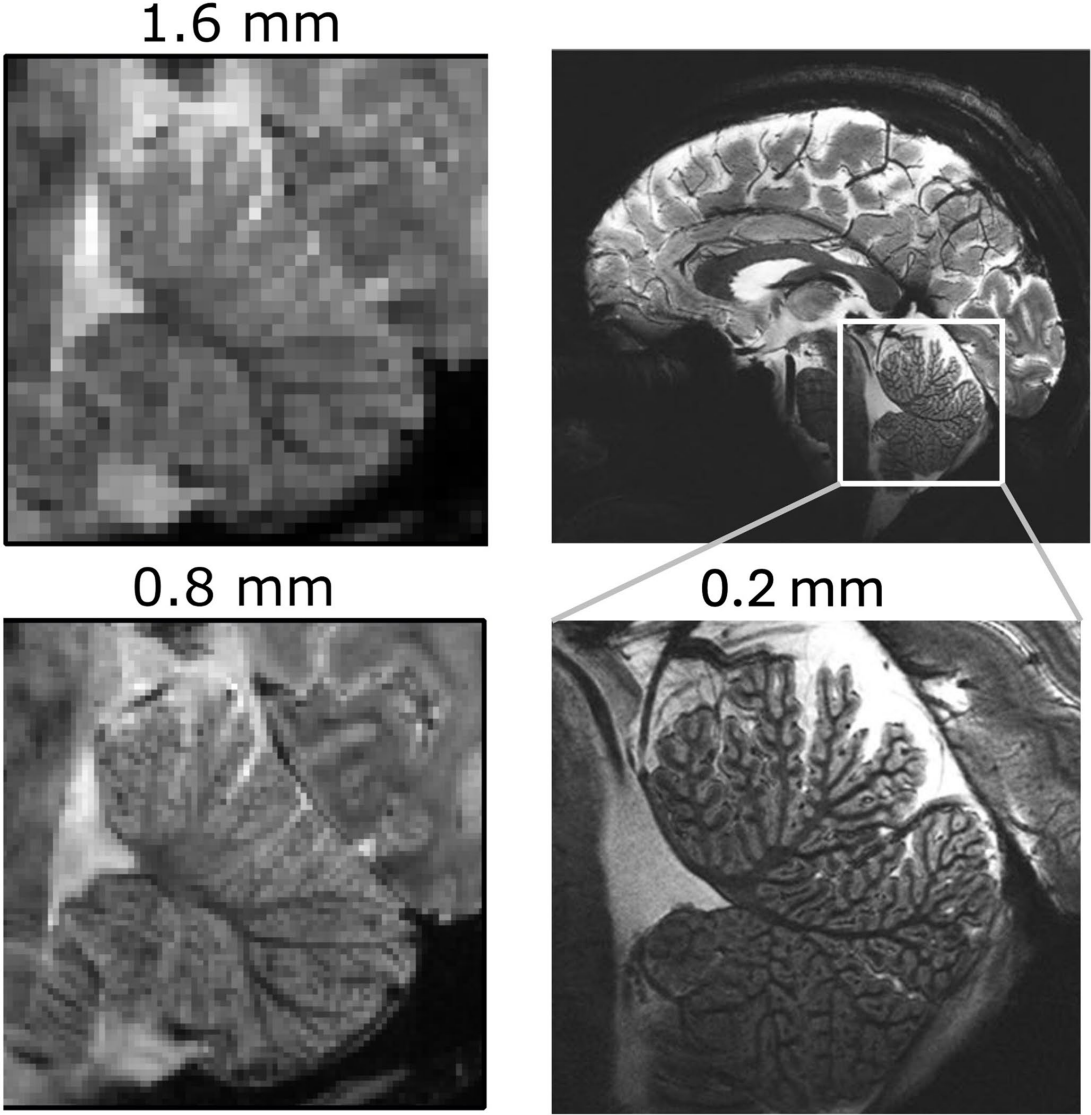
Importance of resolution in cerebellar imaging. Fine detail of the human cerebellum is only visible at the highest spatial resolution. GRE images acquired at 7T and 11.7T (different participants), bottom-right panel: 0.2x0.2x1 mm resolution. Adapted from [15]

Visualisation of fine structural detail of the human cerebellum benefits from high, submillimeter spatial resolution. A current lack of spatial detail at conventional field strengths is partially responsible for the tendency in the field of cognitive neurosciences to ignore the cerebellum in fMRI studies [9]. Cerebellar imaging is challenging: for a delineation of the cerebellar cortex at the level of individual folia, a spatial resolution of well below the millimeter, even down to 0.2 mm, is required [10]. To approach such resolutions *in-vivo*, researchers and clinicians have turned to UHF-MRI, as the increase in signal-to-noise ratio (SNR) offered by higher field strength can be traded for spatial resolution [11, 12]. Using 7 Tesla (7T) or higher field strengths, the human cerebellum has been successfully visualised *in vivo* in fine detail (Fig. 2) [13–17]. However, cerebro-cerebellar network imaging requires both full brain coverage and functional imaging, while sufficient resolution remains a challenge. The use of UHF-MRI itself additionally comes with its own challenges:

### B_1_^+^-inhomogeneity

Although the increase in main magnetic field strength (B_0_) provides the SNR to support the spatial resolution pursued for cerebellar imaging, a challenge arises in the specific shape of the radio frequency (RF)-field generated by transmit coils (B_1_^+^). An area of low B_1_^+^ is found in the posterior lobes of the cerebellum, specifically affecting the right posterior lobe due to the typical human anatomy and coil design [18]. For general neuroimaging, sequence adaptations or new acquisition strategies may provide a viable alternative [13, 19], but for larger heads and specifically for the most inferior regions of the posterior cerebellar lobe, the signal and contrast-to-noise ratio (CNR) may still be affected by the low local B_1_^+^. Although the design of cerebellum-specific coils can provide much higher SNR in the cerebellum [20–22], these tend to come at a cost of signal elsewhere, and do not necessarily provide a solution for cerebro-cerebellar network visualisation. The addition of dielectric pads to the standard coils [23], or universal parallel transmission (pTx) solutions, which do not require time-consuming patient-specific preparation steps [18, 24, 25] provide more flexible signal increases for true whole-brain imaging. When done successfully, even higher field strengths can yield high-quality cerebellar images, despite the shorter RF wavelength [15, 26].

### Functional Imaging at 7T

For functional imaging of the cerebro-cerebellar networks, an additional benefit of UHF-MRI is the increased blood oxygenation level dependent (BOLD) sensitivity [27–29]. 7T cerebellum inclusive whole-brain acquisitions at moderate (~ 1–2 mm) resolution yield reliable BOLD maps even in individual participants, increasing the range of tasks or conditions that may be studied in humans [30]. While the sensitivity to physiological noise in fMRI also increases with B_0_ [31], this is not problematic for cerebellar imaging at high spatial resolution [32]. In the DN, the shorter T_2_* and stronger susceptibility effects associated with its high iron content may require specific acquisition parameter choices to be made. A short TE helps to retain signal locally [33]. Multi-echo fMRI may be an appropriate choice for connectivity studies involving tissues with different optimal TEs, because an early echo can help preserve signal in short-T_2_* regions such as the DN, while later echoes can provide stronger BOLD sensitivity in cortical gray matter. The increased sensitivity of 7T to tissue susceptibility differences also benefits quantitative susceptibility mapping (QSM), which is a useful contrast to anatomically delineate the DN and smaller cerebellar nuclei (globose, emboliform and fastigial nuclei) [17, 34].

One technical aspect to consider when starting cerebellar 7T-fMRI is the quality of the B_0_-shim. Susceptibility-induced distortions always affect the echo planar imaging (EPI) used in typical fMRI acquisitions, and these are more severe if the field homogeneity over the cerebellar area is poorer. Static image-based B_0_ shimming is the current standard in the field [35]. Further improvements in field homogeneity and image quality could be expected from dynamic B_0_ measures, local shim coils or dedicated software tools for B_0_ shimming [36, 37].

### Diffusion MRI at 7T

Visualising the structural component of cerebro-cerebellar networks using diffusion MRI is challenging at 7T, especially at the spatial resolution desired in cerebellar imaging [38]. At UHF, increased SNR may reduce uncertainty in fitted diffusion metrics such as fractional anisotropy (FA) and principal eigenvectors and support higher-resolution acquisitions, which could be valuable for resolving small cerebellar structures such as the DN [39, 40]. However, these benefits are counterbalanced by the shorter local T_2_ and T_2_^*^, increased B_1_^+^ inhomogeneity, and EPI-related distortions.

Nevertheless, advances in coil design and pTx techniques may prove beneficial, especially in low- B_1_^+^ areas such as the cerebellum [41, 42]. New hardware developments at 7T including gradient coils are particularly encouraging for diffusion MRI and may also bring advantages to cerebellar diffusion imaging [43]. The multi-synaptic connections to the cerebral cortex, through the dentate and thalamus or back to the cerebellum through the pons, pose a serious challenge for cerebro-cerebellar network visualisations using diffusion MRI [44]. For a full description of the cerebro-cerebellar networks it is essential to look beyond the cerebellum and cerebrum to the brainstem, which also shapes the cortical functional architecture [45], reflecting the closely linked origin and evolution of the cerebellum and brainstem. So far, interactions between the human brainstem and cerebellum remain terra incognita [46].

## Challenges in Cerebellar Image Analysis

The improved spatial resolution available at 7T drives a need to optimize image analysis for accurate cerebro–cerebellar network mapping. The cerebellum’s intricate foliation and distinctive fine-scale organization bring additional challenges for analysis [47]. Analysis pipelines are required that preserve and exploit the submillimeter detail and cope with the different noise behavior in submillimeter data, as well as specialized segmentation strategies tailored to cerebellar anatomy. Appropriate representation frameworks, such as foliation-preserving surface or flatmap models, along with cerebellum-specific templates, are necessary for accurate inter-subject normalization. Another challenge lies in the need for analysis pipelines that treat the cerebellum and cerebrum as a single, integrated network rather than as independent targets of study.

The spatial resolution necessary to resolve fine cerebellar structure comes at the cost of reduced voxel-wise SNR and increased sensitivity to motion, as well as residual artifacts from parallel-imaging reconstructions. This resolution–SNR/artifact trade-off poses consequences for quality control and preprocessing methods [48]. Although spatial smoothing effectively increases SNR, it is generally avoided in submillimeter imaging to reduce mislocalization [49]. Promising alternatives include anatomically informed smoothing [50], use of the signal phase in image reconstruction [48] or a thermal noise removal such as NORDIC [51]. Data acquired at higher temporal resolution are expected to benefit further from preprocessing approaches that explicitly model physiological contamination [52, 53]. Thus, use of a dedicated, noise-conscious pipeline is highly recommended for cerebellar imaging.

### Cerebellar Segmentation for Cerebro–cerebellar Network Definition

To study cerebro–cerebellar networks, robust segmentation pipelines are required for both the cerebrum and the cerebellum. The segmentation modules of widely used neuroimaging packages [54–56], primarily focus on the neocortex and may therefore fall short in capturing cerebellar structure with the required precision. Cerebellum-specific tools provide automated cerebellar isolation, and lobular parcellation [57–59]. However, these methods generally do not resolve the fine anatomical details to the level of the individual folia. Recent work has addressed these limitations by combining UHF-MRI with automated, topology-preserving cerebellar segmentation pipelines capable of retaining fine cortical detail at the mesoscale *in vivo* [10, 16].

Segmentation of the DN, the principal output hub of the cerebellum and an essential part of the cerebro-cerebellar network, is typically based on QSM data rather than the T_1_-weighting used to isolate the cerebellar cortex. Recently, deep-learning–based segmentation pipelines have been developed to isolate the DN, making it feasible to include the DN as a node in cerebro–cerebellar network analyses [60, 61]. In parallel, whole-brain analysis approaches such as NextBrain have been proposed to provide integrated, high-resolution segmentation across the entire brain [62]. Without such fine-grained segmentation frameworks, cerebro–cerebellar network models risk being asymmetrically defined.

### Surface Reconstruction of the Cerebellar Cortex

**Fig. 3:**
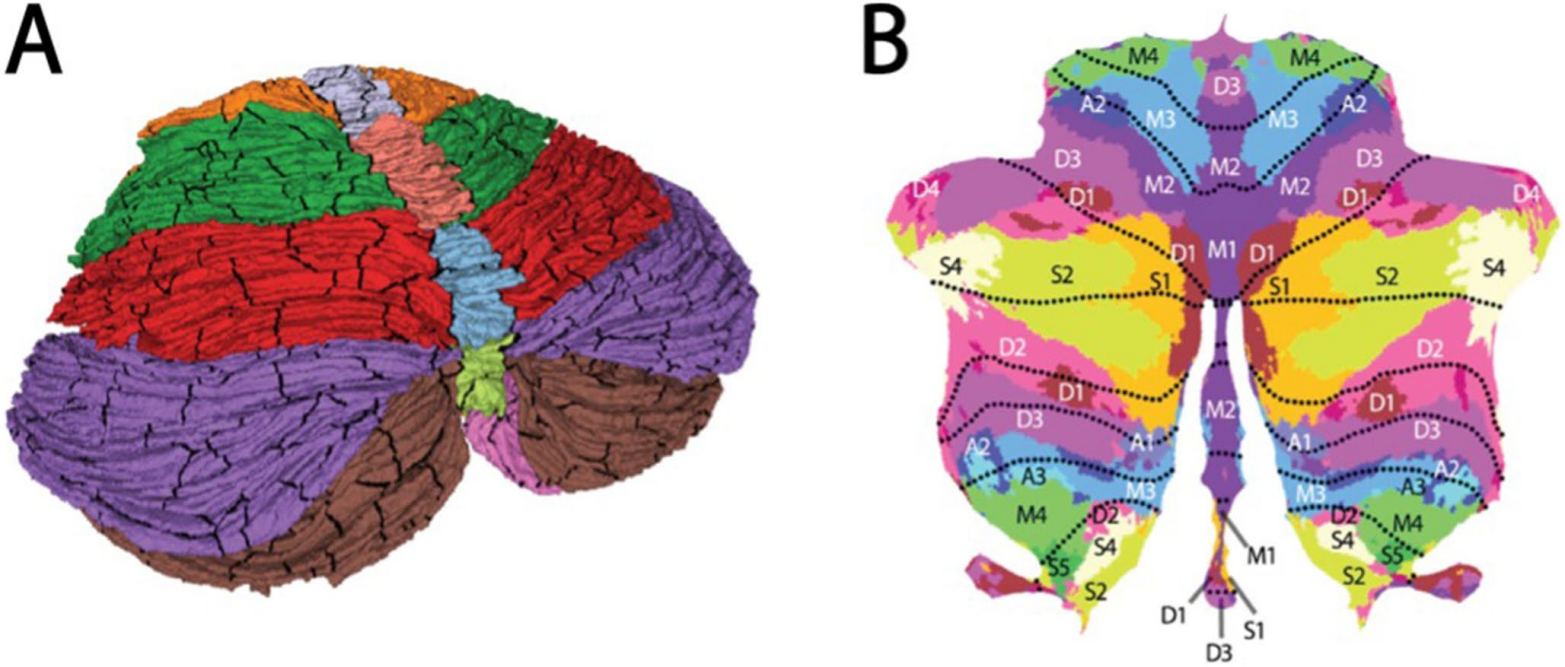
Cerebellar surface and atlas examples. (**A**) Cerebellar surface reconstruction derived from a postmortem scan, figure from [63] (**B**) Functional atlas with medium-level granularity from [66]

Reconstructing the cerebellar surface is considerably more complex than reconstructing the neocortex due to high intrinsic curvature and extreme foliation. Until recently, no computational reconstruction had resolved the full complement of individual cerebellar folia (Fig. 3A) [63]. Sereno et al., [10] first achieved this in postmortem 9.4T MRI, extending the FreeSurfer package [54] to reconstruct, unfold, and flatten the entire cerebellar cortical surface at folia-level detail. This framework also enabled detailed reconstruction of the deep cerebellar nuclei (DCN), including the DN, thereby providing anatomically precise definitions of cerebellar output structures.

Building on this foundation, recent 7T frameworks have enabled high-fidelity *in vivo* cerebellar surface reconstruction, recovering approximately 90% of the postmortem surface area within clinically feasible acquisition times [16]. Together, these advances enable practical integration of cerebellar surfaces into cerebro–cerebellar network analyses.

### Cerebellum-specific Atlas Frameworks

Inspection of functional network analysis results requires atlases and a standard space. Task-evoked or resting-state BOLD responses can then be projected onto cerebellar flatmaps and compared to functional atlases. For cerebellar images, the SUIT toolbox provides an atlas template and flatmap deformation tailored to the human cerebellum [57]. This enables more accurate alignment of lobular anatomy and functional parcellation compared to traditional whole-brain normalization methods, despite remaining limitations in fissure registration [47, 64]. King et al. introduced a task-based multi-domain functional parcellation in SUIT space spanning motor, cognitive, and affective domains [65]. More recently, a hierarchical atlas with three nested levels of granularity has been introduced (Fig. 3B) [66]. These tools allow cerebellar BOLD patterns to be interpreted in terms of specific functional territories and their putative counterparts in large-scale cerebral networks [47, 65, 66].

Despite their utility, flatmap-based atlases necessarily simplify complex three-dimensional foldings and the fine fissural organization of the cerebellum. Lyu et al., [67] introduced a surface-based cerebellar atlas derived from high-resolution MRI that adheres closely to fissural boundaries when delineating cerebellar subregions. Even more recently, a digital atlas based on high-resolution cerebellar surface reconstructions has been developed, enabling accurate delineation of the subfolial contours [63]. These advances improve anatomical precision and incorporate structural connectivity information, potentially offering a refined framework within which functional parcellations can be interpreted.

Together, these advances can help tackle a longstanding debate in the field: how a cerebellar cortex with remarkably uniform microstructure can give rise to distinct functional domains across motor, cognitive, and affective tasks [68]. In addition, the exploration of mesoscale information on the cerebellar surface, whether structural, vascular or functional can be further refined [16, 69, 70]. Two main types of Purkinje cells, subdivided based on protein expressions and intrinsic firing frequencies, have been identified in preclinical work and may reflect functional subunits [71]. For successful clinical translation, 7T MRI can be used to perform structural profiling in patients with different cerebellar disorders and provide functional read-outs that are necessary to design and test targeted interventions.

## Cerebellar Nodes as Targets for Circuit-specific Neuromodulation

Although 7T provides detailed cerebellar maps, current neuromodulation studies have not yet exploited them. UHF-MRI at 7T is increasingly utilized to enhance precision of deep brain stimulation (DBS) targeting, as it offers superior spatial resolution, SNR, and tissue contrast for visualizing the deep brain nuclei including the DCN and their associated networks. Multi-institutional recommendations, drawing on experience from more than 1,000 surgical procedures [72], emphasize the clinical value of 7T MRI for patient-specific surgical planning and postoperative programming. Optimized acquisition and postprocessing techniques, including QSM, fast gray matter acquisition T_1_ inversion recovery (FGATIR), and advanced diffusion imaging of tracts such as the dentato-rubro-thalamic tract, enable improved delineation of midbrain targets including the subthalamic nucleus, globus pallidus internus, and thalamus while mitigating field distortions and artifacts.

The principles of high-resolution, individualized anatomical, and connectivity-guided targeting apply equally to cerebellar neuromodulation. The cerebellum provides accessible nodes within cerebro-cerebellar circuits that can be modulated non-invasively to influence distributed cerebral networks. Rohira and colleagues demonstrated that MRI-neuronavigation, combining structural T1-weighted imaging with the SUIT template, significantly outperforms traditional scalp landmark-based approaches (e.g., inion-lateral or inion-mastoid) for cerebellar transcranial magnetic stimulation (TMS) [73]. Electric field modeling across 20 participants confirmed that 7T based neuronavigation-guided coil placement produces stronger E-fields in intended cerebellar territories (such as Crus II) and reduces unintended spill-over into adjacent regions (e.g., occipital cortex), with effects modulated by coil type (figure-of-8 versus double-cone) [73].

### Translational Implications of High Resolution Cerebellar Targeting

Although many contemporary cerebellar TMS studies continue to rely on 3T MRI for functional connectivity mapping and targeting, UHF-MRI provides clear advantages for refinement. A 7T-inclusive protocol has been implemented to examine repetitive TMS effects on fatigue in multiple sclerosis, incorporating high-resolution structural, functional, and spectroscopic imaging [75]. UHF-MRI delivers enhanced BOLD sensitivity for individualized resting-state network mapping, superior visualization of cerebellar cortical subregions, and direct assessment of the DCN. Anteraper and colleagues [76] highlight how 7T MRI can advance functional parcellation of the DN and support targeted non-invasive stimulation of specific cerebro-cerebellar territories to refine therapeutics for psychotic disorders.

Collectively, UHF structural and functional imaging, advanced nuclei segmentation, and connectivity mapping enables more reliable subject-specific targeting, better verification of engagement of intended cerebro-cerebellar networks, and a hopeful minimization of off-target effects. In translational contexts such as schizophrenia, multiple sclerosis, post-stroke recovery, and other disorders involving disrupted cerebro-cerebellar circuitry, integration of 7T MRI promises to improve reproducibility, mechanistic understanding, and clinical efficacy of cerebellar neuromodulation [77]. Future progress will benefit from standardized UHF-inclusive pipelines that maintain compatibility with widely available 3T systems for broader clinical translation.

## Cerebellar Circuitry Across the Lifespan

Early cerebellar damage has long been implicated in a spectrum of developmental cognitive and affective disorders [78, 79]. In the brain of infants born preterm, the cerebellum is particularly susceptible to hemorrhage resulting in motor delays and cognitive impairments [80]. Compared to other brain injuries, however, cerebellar hemorrhage has largely been under-studied in this population, leaving a significant gap in the mechanistic understanding of, and developing therapies for, preterm cerebellar damage. Importantly, early cerebellar damage is known to result in altered development of functionally connected regions in the cerebral cortex [81–84]. For example, patients with right cerebellar damage may develop a hypoplastic left cerebral cortex. Prior studies report ten-times higher rates of autism spectrum disorder (ASD) and five-times higher rates of attention deficit hyperactivity disorder (ADHD), as well as increased likelihood of epilepsy, ataxia and anxiety, and mood disorders in preterm births, posing a significant healthcare burden [85]. On the other hand, potentially because of cerebellar reserve, the cerebellum’s remarkable capability to reconfigure, compensate, and restore itself after pathological injury, speaks to developmental strength. Leveraging the strength of 7T, specialized head coils, and motion-corrected acquisitions, investigating this dichotomy can usher new opportunities for investigating and identifying preventive measures for developmental arrest, intervening at possible critical periods, and limiting potential long-term consequences of cerebellar damage.

Adolescence has the second fastest rate of neurodevelopment after infancy, yet cerebro-cerebellar circuitry remains under-examined in this population. Anxiety disorders are the most common mental illnesses among adolescents, with a median age of onset at only 11 years old. Early detection is key as anxiety disorders may predispose adolescents to other psychiatric disorders. Resting state 3T-fMRI studies reveal cerebro-cerebellar functional connectivity alterations in adolescent anxiety and point to the DN as a potential target for disease prediction [74].

Considering cerebellar involvement in higher-order cognitive processes, research efforts that specifically target the cerebellum to enhance cognitive reserve may play a critical role in delaying the onset and progression of Alzheimer’s disease (AD) and related dementias (ADRD). By modulating cerebellar activity—through approaches such as neurostimulation or cognitive training—cerebro-cerebellar networks that compensate for early pathological changes can be strengthened. Importantly, such investigations can also contribute to the identification of early biomarkers and structural or functional signatures of cognitive decline and neurodegeneration. Changes in cerebellar connectivity, volume, or activity patterns may serve as sensitive indicators of preclinical or prodromal stages of AD/ADRD. Thus, targeting the cerebellum not only offers a potential therapeutic pathway to build resilience against neurodegeneration but also enhances our ability to detect and monitor disease progression at earlier, more actionable stages. The likelihood of early-onset AD and related dementias among adults with ASD is 2.6x higher than in the general population [86], which calls for lifespan studies in individuals with ASD.

Deep cerebellar nuclei such as the DN, neuronal bodies embedded in cerebellar white matter, link the cerebellum to cerebral, thalamic and basal ganglia circuits [87, 88] but their role in AD is only emerging [89]. Employing 7T-fMRI can improve the understanding of the cerebello-dentato-thalamo-cortical circuitry leading to improvements in sensitivity and specificity of currently available neurophysiological instruments and potentially allow early detection method improvement [76].

## An Outlook From Postmortem Imaging for *In-vivo* 7T MRI

Postmortem MRI of the human cerebellum can offer a unique outlook into ultra–high-resolution structural and microstructural mapping. In postmortem specimens, the absence of motion and possibility of hours-to-days long scanning enable images with voxel sizes around 0.1 mm^3^, revealing cerebellar fissuration patterns, cortical lamination, and gray-white matter architecture in far greater detail than is feasible *in vivo* [90, 91]. Multimodal acquisitions help segment small subregions in the extra-cerebellar structures of the cerebro-cerebellar networks, including the parvocellular and magnocellular portions of the red nucleus, accessory olive nuclei, and pontine nuclei [92]. Parvocellular red nucleus links to cerebral cortical M1 and magnocellular links to motor and associative cortical areas and DN. Diffusion-weighted imaging at such resolutions allows tractography of major cerebellar white-matter pathways and even intracortical fibres within the cerebellar cortex, to visualize, using micro-tractography, the fibres between the cerebellum and pontine nuclei and red nucleus [93, 94]. A major advantage of postmortem imaging is the further possibility of histological validation, allowing correlations of MR signal to be made with myelin content, cell density, and microvascular architecture at the level of individual folia [90, 95, 96].

**Fig. 4:**
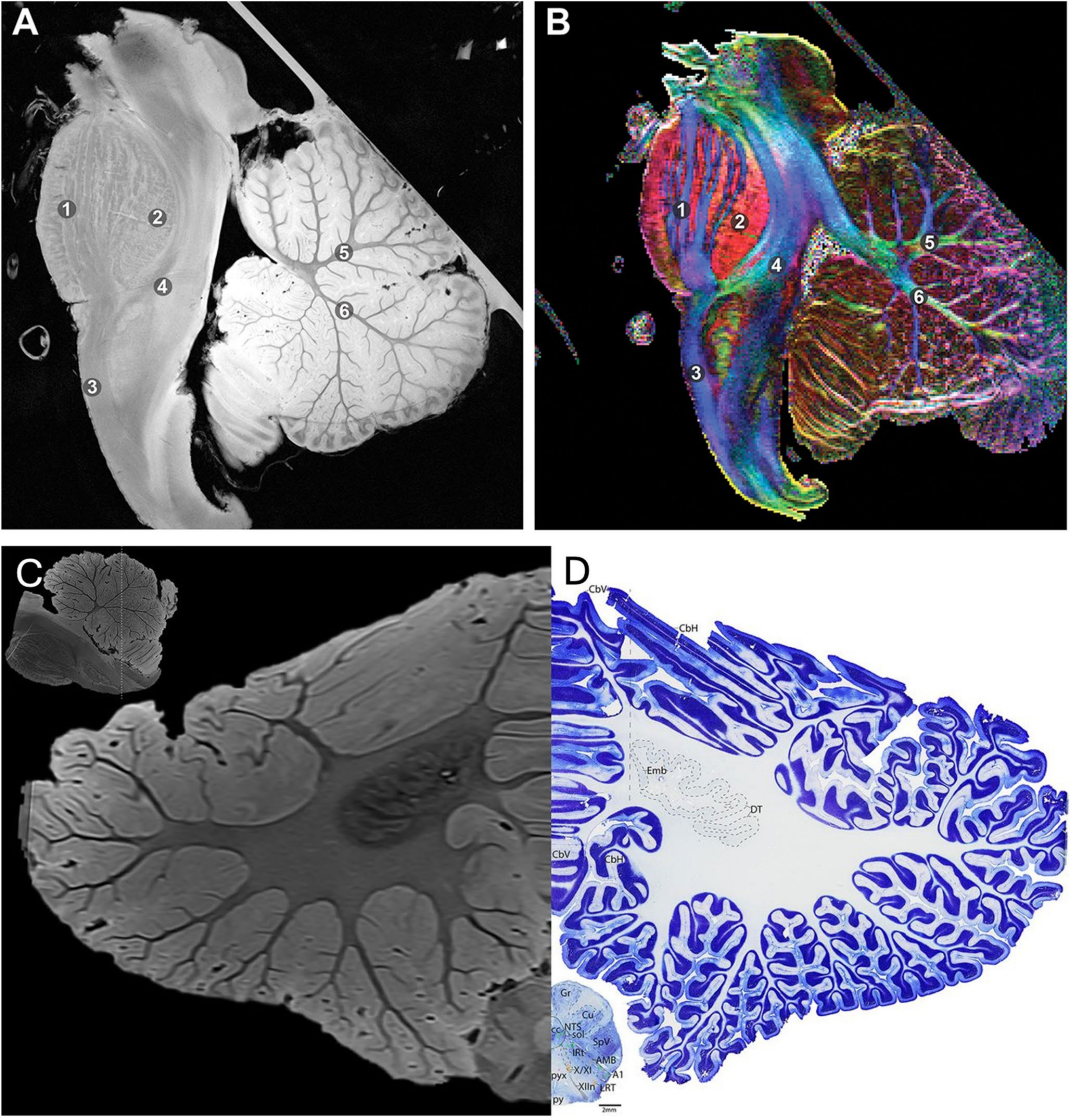
Post-mortem 7T cerebellar MRI. Exquisite detail in anatomy (**A**) and structural connectivity (**B**), which is currently unattainable in-vivo. Combinations of 7T data with histological staining provide added insight in the underlying tissue microstructure (**C**, **D**). Figures taken with permission from [94] (**A**, **B**) and [103] (**C**, **D**)

Formalin fixation, however, modifies MRI contrast and must be taken into account when interpreting postmortem cerebellar data [97–100]. Changes in T1 and T2 relaxation times reflect chemical cross-linking, temperature differences, and altered water mobility [101, 102]. Diffusion indices are also affected, although in a somewhat more moderate fashion; basic patterns of anisotropy in fine white-matter bundles remain clearly detectable (Fig. 4AB) [94].

To link postmortem and *in vivo* MRI, recent quantitative protocols combined high-resolution postmortem MRI with standardized *in vivo* acquisitions in the same donors, enabling direct comparison of relaxation times, diffusion metrics, and morphometry across the two states [96]. Histology-registered postmortem datasets are used to anchor MRI contrasts to underlying cyto- and myeloarchitecture, creating a three-way mapping between *in vivo* MRI, ex vivo MRI, and microscopy (Fig. 4CD) [103]. This is particularly valuable in the cerebellar cortex, where thin laminae and tightly folded folia pose challenges for purely in vivo work [90, 95] .

### Deep-leaning Helps Bridge MRI and Histology

Deep learning has recently emerged as an important tool to strengthen the bridges between postmortem and *in vivo* imaging. Convolutional neural networks trained on co-registered multi-contrast MRI and histology can infer voxelwise histological staining intensities from MRI signals, effectively providing ‘*virtual histology’* for axons and myelin [104]. Similar approaches have been used to translate high-resolution ex vivo MRI into histology-like contrasts and to generate *in* *vivo*-like images from postmortem datasets [105, 106]. More integrated pipelines that combine *in vivo* MRI, in vivo optical imaging, and ex vivo super-resolution microscopy at near-voxel accuracy allow deep learning models to predict disease-relevant histopathology from MRI alone, suggesting a route by which cerebellar postmortem data could inform interpretation of *in vivo* human scans [104]. As high-resolution postmortem atlases of the human cerebellum and whole brain expand and are integrated with deep learning-based virtual histology, the role of postmortem MRI as a bridge between microstructure and *in vivo *cerebellar imaging is likely to grow substantially.

## Methods and Materials: Scope, Literature Identification and Writing Process

We conducted a targeted literature search in online scientific databases including PubMed and Google scholar. Searches combined ultra-high-field terms (“7T”, “ultra-high field”) with cerebellar and technique-specific keywords (e.g.“cerebro-cerebellar connectivity”, “diffusion imaging human cerebellum”, “quantitative susceptibility mapping”). Only English-language publications were considered. Titles and abstracts were screened for relevance to cerebro–cerebellar network mapping at 7T, with emphasis on acquisition advances, preprocessing, and denoising, cerebellum-specific normalization or parcellation. When relevant, full texts were reviewed and additional papers were identified via backward/forward citation tracking. Authors discussed the emerging themes and drafted conclusions during regular meetings between August 2025 and March 2026.

## Conclusion and Outlook

For future experiments on cerebellar MRI at UHF, in terms of the acquisition, it is recommended to employ a form of pTx to ensure high local SNR. True whole-brain analysis tools are required for cerebro-cerebellar network analysis with such segmentation and visualisation tools now starting to become available. We can draw on knowledge gathered from postmortem work to identify imaging and treatment targets in-vivo, such as substructures within the cerebellum including the DCN.

With current developments, UHF MRI can provide a window to biology though myelin- and iron-sensitive contrast at submillimeter resolution. In future, these developments can help access cerebellar substructures characterised by myelination differences and provide further insight into the cerebro-cerebellar networks. Additionally, continued progress in specificity and resolution will allow the inclusion of other nodes in the cerebro-cerebellar networks, including those in the brainstem, pons and DCN.

For the cerebellum to truly become part of standard neuroimaging, there is an urgent need for (i) increasingly robust and reliable cerebellum-inclusive research methodologies, protocols, and collaborative platforms for researchers, including standardized imaging at UHF; (ii) neuroimaging analysis tools that ensure the cerebellum is incorporated routinely into whole-brain cognitive and disease models; (iii) imaging of the cerebro-cerebellar contributions in the context of cerebellar reserve and disease; (iv) real-world translation of biomarkers to serve as outcome measures in clinical trials for neurodegenerative diseases; (v) inclusion of the cerebellum as a novel treatment option for non-invasive transcranial stimulation. Only through coordinated effort will the cerebellum move from a frequently overlooked structure to a routinely interrogated node in systems-level models of brain function and disease.

## Data Availability

No datasets were generated or analysed during the current study.
